# Luteolin suppresses gastric cancer progression by reversing epithelial-mesenchymal transition via suppression of the Notch signaling pathway

**DOI:** 10.1186/s12967-017-1151-6

**Published:** 2017-02-27

**Authors:** Ming-de Zang, Lei Hu, Zhi-yuan Fan, He-xiao Wang, Zheng-lun Zhu, Shu Cao, Xiong-yan Wu, Jian-fang Li, Li-ping Su, Chen Li, Zheng-gang Zhu, Min Yan, Bing-ya Liu

**Affiliations:** Department of Surgery, Shanghai Key Laboratory of Gastric Neoplasms, Shanghai Institute of Digestive Surgery, Ruijin Hospital, Shanghai Jiao Tong University School of Medicine, Shanghai, 200025 People’s Republic of China

**Keywords:** Gastric cancer, Luteolin, Notch1, β-Catenin, Apoptosis, EMT

## Abstract

**Background:**

Gastric cancer (GC) is one of the most malignant tumors and the second leading cause of cancer-related deaths in the world. Luteolin, a flavonoid present in many fruits and green plants, suppresses cancer progression. The effects of luteolin on GC cells and their underlying mechanisms remain unclear.

**Methods:**

Effects of luteolin on cell proliferation, migration, invasion, and apoptosis were examined in vitro and in vivo by cell counting kit-8 (CCK-8), transwell assays, and flow cytometry, respectively. Real-time reverse transcription polymerase chain reaction (RT-PCR) and Western blots were performed to evaluate Notch1 signaling and activation of epithelial-mesenchymal transition (EMT) in GC cells treated with or without luteolin. Immunohistochemistry was performed to examine proliferation and Notch1 expression in xenograft tumors.

**Results:**

Luteolin significantly inhibited cell proliferation, invasion, and migration in a dose-dependent and time-dependent manner and promoted cell apoptosis. Luteolin reversed EMT by shrinking the cytoskeleton and by inducing the expression of epithelial biomarker E-cadherin and downregulating the mesenchymal biomarkers N-cadherin, vimentin and Snail. Furthermore, Notch1 signaling was inhibited by luteolin, and downregulation of Notch1 had similar effects as luteolin treatment on cell proliferation, migration, and apoptosis. In addition, luteolin suppressed tumor growth in vivo. A higher expression of Notch1 correlated with a poor overall survival and a poor time to first progression. Furthermore, co-immunoprecipitation analysis revealed that activated Notch1 and β-catenin formed a complex and regulated cell proliferation, migration, and invasion.

**Conclusions:**

In this study, GC progression was inhibited by luteolin through suppressing Notch1 signaling and reversing EMT, suggesting that luteolin may serve as an effective anti-tumor drug in GC treatment.

## Background

The incidence of cancer is higher worldwide due to various factors such as smoking, environmental pollution, obesity and aging. Gastric cancer is the fourth most common cancer and the second leading cause of cancer-related deaths in the world [1]. It is the leading cause of tumor-related deaths among males in China [2]. However, effective GC treatment is absent and resistance to chemotherapy is one of its most crucial obstacles, particularly in advanced GC. Due to a lack of validated screening programs, most GC patients are diagnosed at a late stage, leading to a high mortality, in developing countries [3, 4]. Therefore, it is necessary to identify mechanisms underlying GC development as well as design novel drugs for its treatment. Artemisinin, which is isolated from a Chinese herb, suppresses tumor development by causing cell cycle arrest and inducing apoptosis in cancer cells [5, 6]. Luteolin is a flavonoid present in many fruits and green plants, and has the ability to suppress cancer progression [7, 8], which indicates that it may be used as a drug for the treatment of tumors.

Notch signaling is implicated in a majority of cancers for promoting the malignant phenotype by inducing cell proliferation, metastasis, drug resistance, and inhibiting apoptosis [9–12]. Ligand binding to Notch, which is a single-pass transmembrane receptor, leads to its cleavage and release of the Notch intracellular domain (NICD), which translocates to the nucleus and interacts with transcription factor RBPJ to regulate cellular functions [13–15]. The Wnt/β-catenin pathway is conserved across species [16] and regulates tissue development in embryos and tissue maintenance in adults. Aberrant activation of Wnt/β-catenin promotes the progression of a variety of cancers due to uncontrolled cell proliferation and growth [17, 18]. There is a crosstalk between the Notch and Wnt/β-catenin signaling pathways in many cell types for regulating cell proliferation and migration during development [19, 20]. However, this crosstalk may cause synergistic or antagonistic effects depending on the context [21, 22], and its status in GC remains unclear.

Epithelial-mesenchymal transition (EMT) is not only a physiological process but also a pathological process that regulates cell phenotype and functions during embryogenesis as well as tumor development [23–25]. Morphological changes due to EMT and effects of the tumor microenvironment cause resistance to therapy in many cancers through a number of signaling pathways [26–28]. Notch signaling-induced EMT is a key factor implicated in tumor metastasis [29–31]. Therefore, we addressed the relationship between Notch and EMT in GC progression.

In order to identify the mechanisms underlying GC development as well as effective treatment methods, we studied the therapeutic effect of luteolin on GC and its potential molecular mechanisms of action.

## Results

### Luteolin inhibits the proliferation and colony formation ability of GC cells

**Fig. 1:**
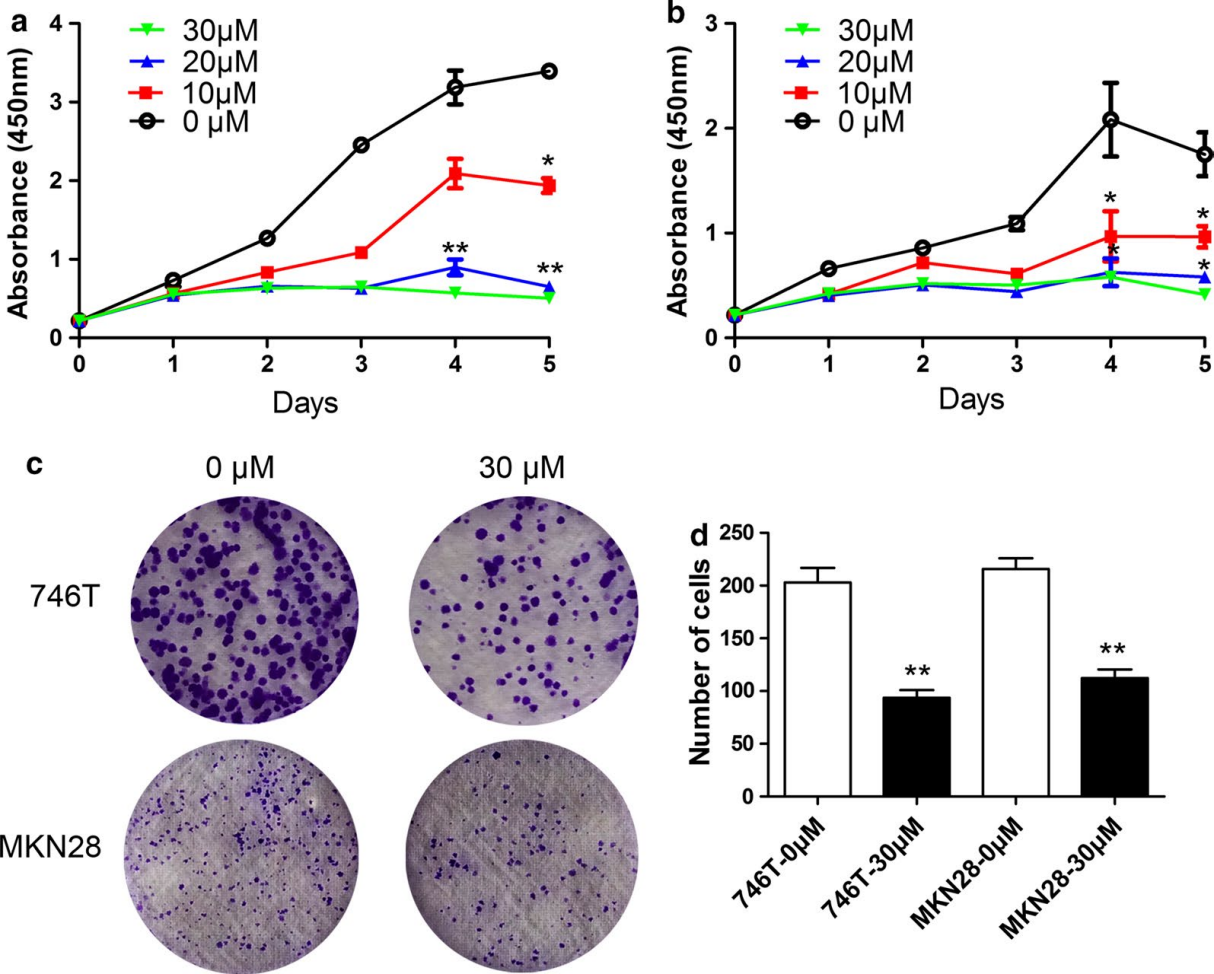
Effects of luteolin on proliferation and colony formation ability in GC cells. **a** The proliferation of Hs-746T GC cells was inhibited upon luteolin treatment compared with the control group. Cell proliferation curves indicated that luteolin suppressed the growth of GC cells in a dose- and time-dependent manner. The significant inhibited effect on cell growth by luteolin was observed at 4th and 5th day after luteolin treatment. The results of 4th and 5th day were compared to that in their control groups using the Student’s t test. There was no statistical significance at 4th day after 10 μM lueolin treatment compared with 0 μM luteolin, but a statistical significance at 5th day. Both 20 and 30 μM luteolin resulted in a statistical significance at 4th and 5th day. **b** The proliferation of MKN28 GC cells was inhibited upon luteolin treatment compared with the control group. The results of 4th and 5th day were compared using the Student’s t test. There was a statistical significance at 4th and 5th day after lueolin treatment compared with 0 μM luteolin. **c** Luteolin significantly reduced the colony formation ability of GC cells. **d** Number of colonies in control and luteolin-treated groups in two GC cell lines. Results are the means of three independent experiments. *P < 0.05, **P < 0.01

Hs-746T and MKN28 GC cells were cultured with 0, 10, 20 and 30 μM luteolin. CCK-8 assay was performed every 24 h, and results showed that proliferation of GC cells were effectively inhibited by luteolin in a dose- and time-dependent manner (Fig. 1a, b). Moreover, luteolin treatment also significantly reduced the number of colonies compared with the control group (for Hs-746T, *P* = 0.0097; for MKN28, *P* = 0.0014; Fig. 1c, d).

### Luteolin promotes apoptosis in GC cells

**Fig. 2:**
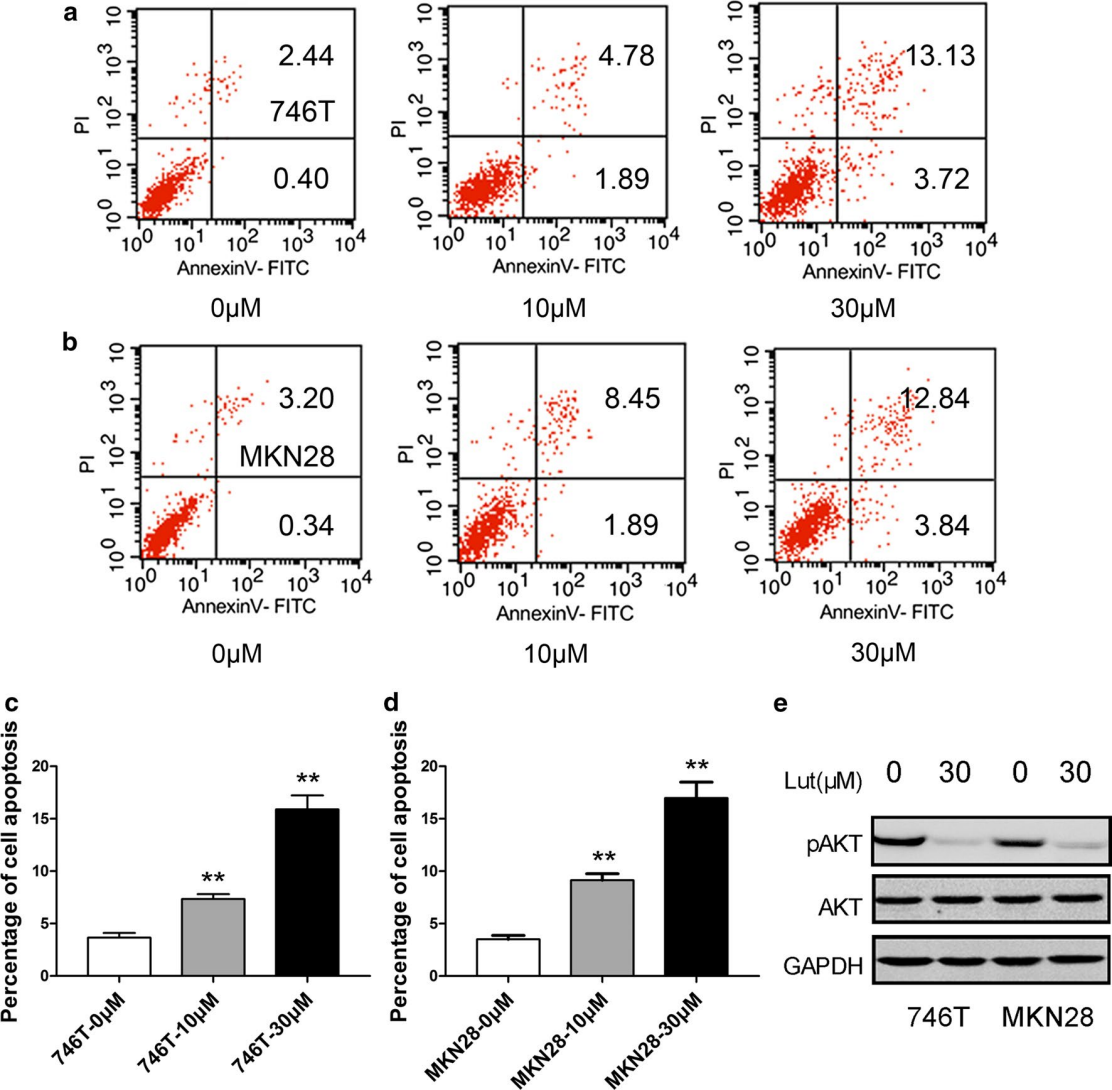
Effect of luteolin on cell apoptosis in GC. **a**, **b** The apoptosis of GC cells was increased upon luteolin treatment compared with the control groups. The percentages of both early and late apoptotic cells in 10 and 30 μM luteolin-treated groups were higher than the control groups. **c**, **d** The *histograms* show the percentage of cell apoptosis in GC cells. **e** Phosphorylation of Akt (Ser-473) was inhibited by luteolin, as observed by Western blot analysis. Results are the means of three independent experiments. *P < 0.05, **P < 0.01

The percentage of early and late apoptosis was increased upon treatment with 10 and 30 μM luteolin compared with the control group (Fig. 2a, b). The proportion of apoptotic Hs-746T (0 vs. 10 μM, *P* = 0.0047, 0 vs. 30 μM, *P* = 0.0009, Fig. 2c) and MKN28 (0 vs. 10 μM, *P* = 0.0014, 0 vs. 30 μM, *P* = 0.0010, Fig. 2d) cells increased in a dose-dependent manner. Since PI3K/Akt signaling is implicated in cell apoptosis in a majority of tumors, we examined the phosphorylated Akt levels in GC cells after treatment with luteolin. The results showed that phosphorylated Akt (Ser-473) was decreased by luteolin treatment (Fig. 2e).

### Luteolin inhibits invasion and migration of GC cells

**Fig. 3:**
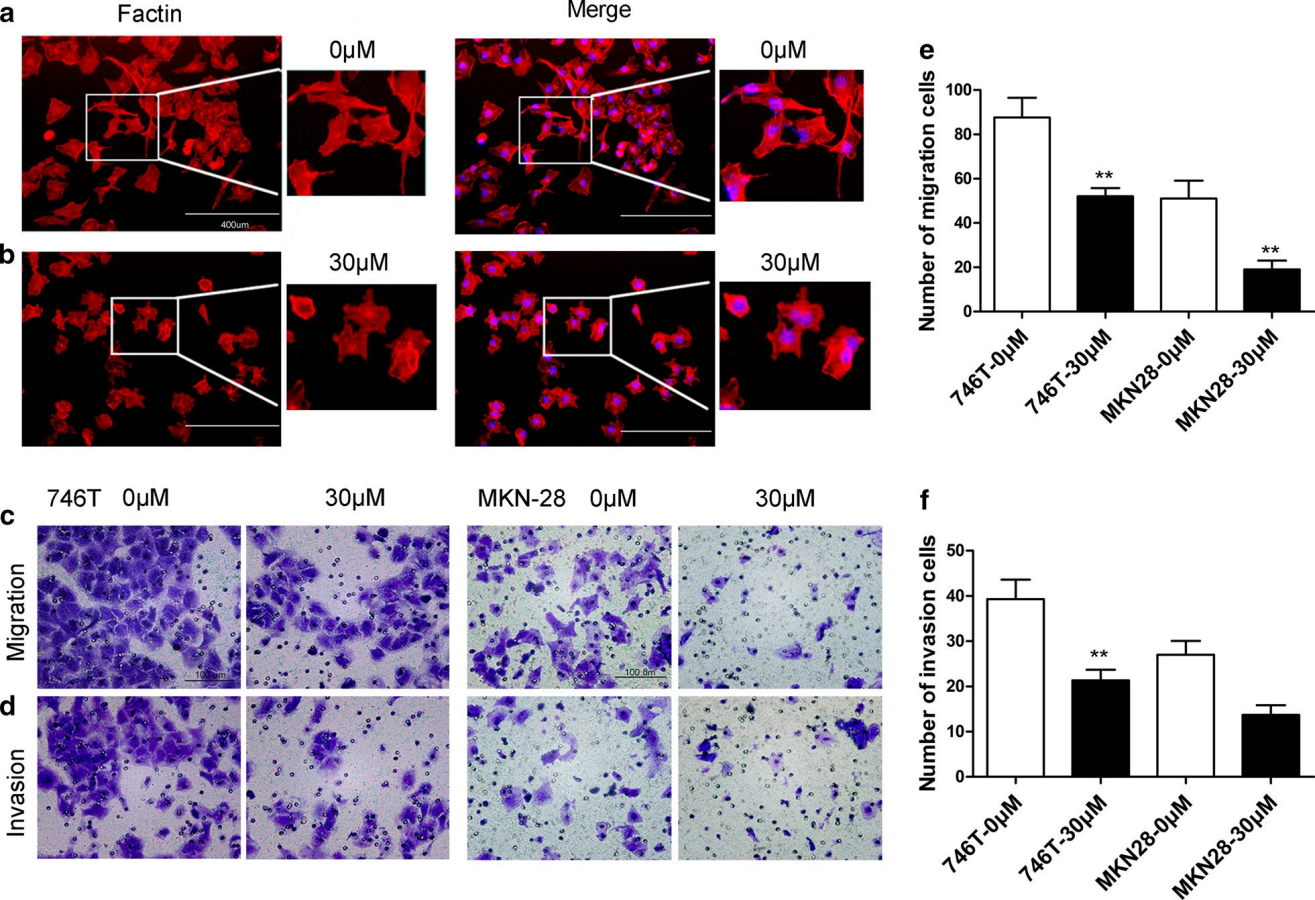
Effects of luteolin on cytoskeleton and motility in GC cells. NCI-N87 GC cells were treated with or without luteolin (30 μM) for 24 h, and analyzed by F-actin staining (*Red* F-actin, *Blue* DAPI, 200×). **a** Control NCI-N87 GC cells showed a spindle and fusiform shape, which indicates higher motility. **b** Luteolin treatment (30 μM) for 24 h caused shrinking in NCI-N87 GC cells and a decrease in the number of pseudopodia on the cell surface. **c**, **d** The cell motility was assessed by transwell assays (200×). **e**, **f** The number of migrating and invading cells is quantified. Results are the means of three independent experiments. *P < 0.05, **P < 0.01

NCI-N87 GC cells showed a mesenchymal phenotype, as evidenced by F-actin staining, in the absence of luteolin treatment (Fig. 3a). However, when NCI-N87 cells were treated with luteolin, the cytoskeleton shrank and cell size decreased (Fig. 3b). These findings indicate that luteolin can suppress the motility of GC cells. Transwell assays showed that invasion and migration of GC cells was significantly inhibited by luteolin treatment (*P* < 0.01, Fig. 3c–f).

### Luteolin reverses EMT and suppresses Notch1 signaling in GC cells

**Fig. 4:**
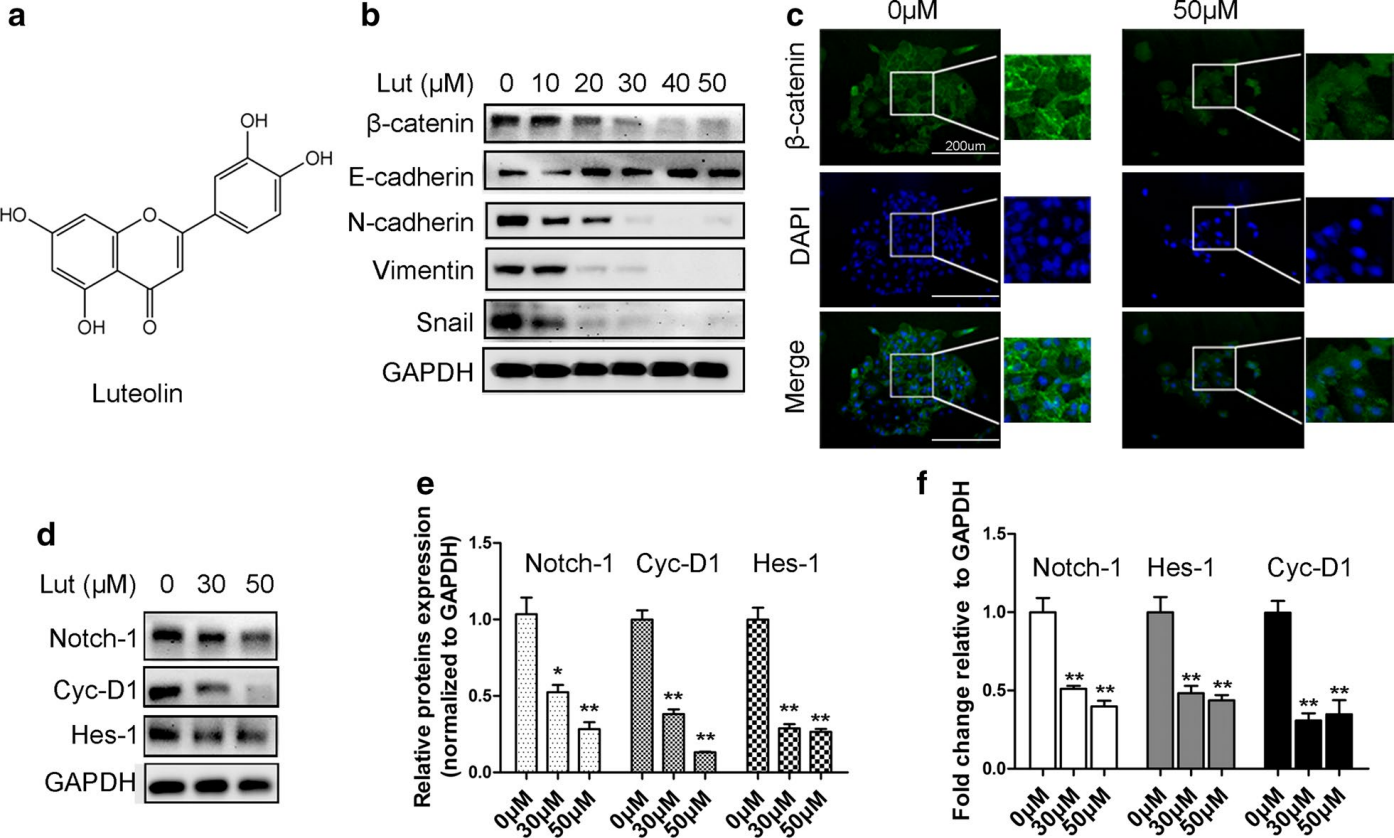
Effects of luteolin on EMT and Notch signaling in GC cells. **a** The chemical structure of luteolin. **b** The protein levels of the EMT markers were assessed by Western blot analysis in GC cells treated with different concentrations of luteolin. Luteolin increased E-cadherin levels and significantly decreased N-cadherin, β-catenin, vimentin, and Snail levels. **c** Immunofluorescence analysis showed that β-catenin was decreased in GC cells upon luteolin treatment (*Green* β-catenin, *Blue* DAPI, 200×). **d** The expression of Notch1, cyclin-D1, and Hes-1 was examined by Western blot analysis in GC cells after treatment with luteolin. **e** *Gray scale* ratio of Notch signaling markers in GC cells. **f** The mRNA levels of Notch targets were evaluated by RT-PCR. Results are the means of three independent experiments. *P < 0.05, **P < 0.01

The remodeling of the cytoskeleton upon luteolin treatment indicated that luteolin may regulate this process by inhibition of EMT in GC cells. We observed that the epithelial biomarker E-cadherin was increased and the mesenchymal biomarkers N-cadherin, vimentin, and Snail were reduced in a dose-dependent manner upon luteolin treatment (Fig. 4b). Luteolin treatment also caused a decrease in β-catenin levels (Fig. 4c). We also found that Notch1, cyclin-D1, and Hes-1 were downregulated due to luteolin treatment (Fig. 4d–f), suggesting that luteolin prevented GC progression by suppressing Notch signaling.

### Luteolin suppresses GC progression via decreasing Notch1 expression

**Fig. 5:**
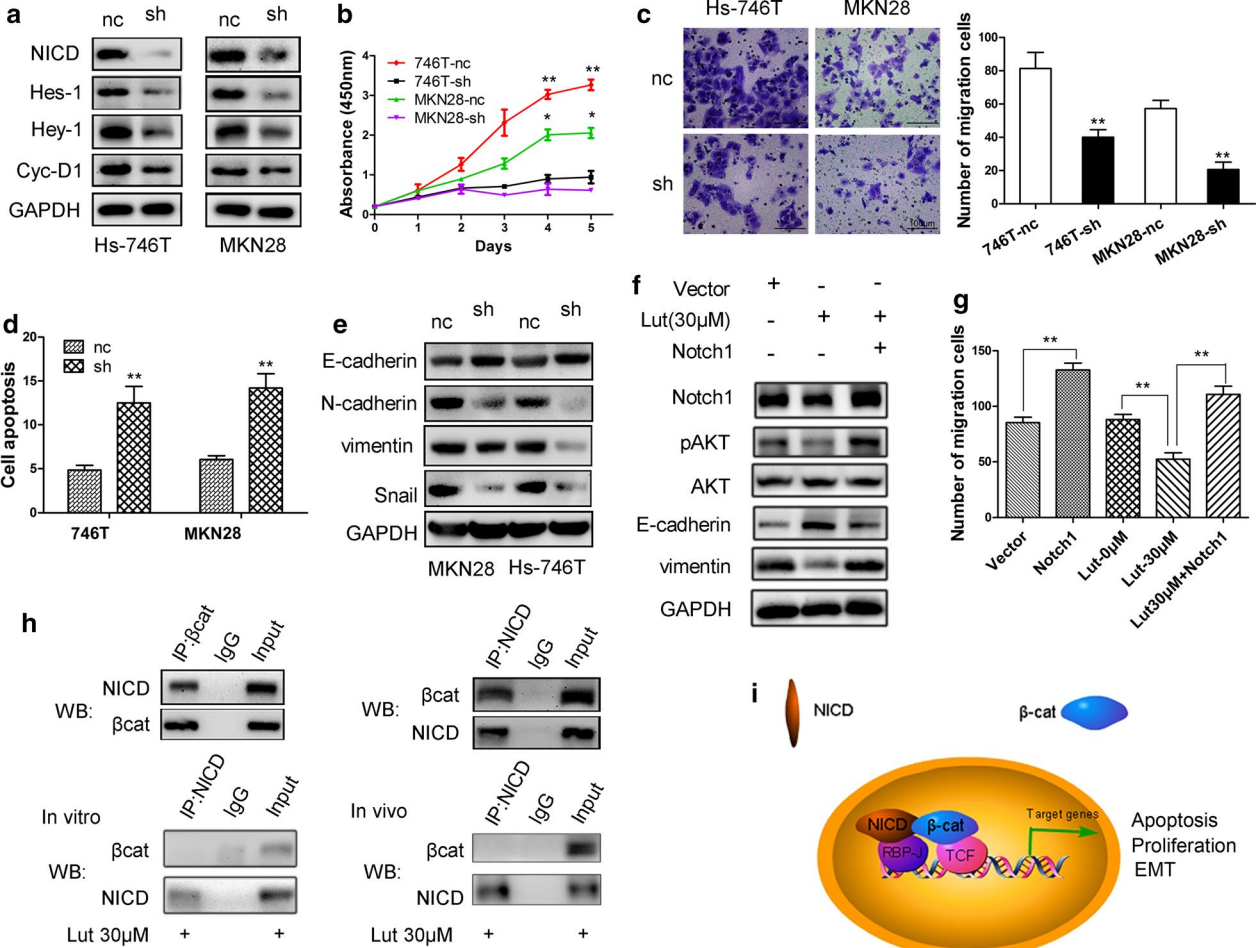
Effects of Notch1 on cell proliferation and EMT in GC cells. **a** The targets of Notch1 signaling were examined by Western blot assay after Notch1 downregulation using a shRNA. **b** Suppression of Notch1 caused inhibition of proliferation in GC cells. **c** The migration ability of GC cells was reduced in Notch1-silenced cells. **d** Suppression of Notch1 induced cell apoptosis. **e** The expression of E-cadherin was increased in Notch1 knocked down GC cells, while in contrast, N-cadherin, vimentin, and Snail expression levels were decreased. **f** Overexpression of Notch1 decreased E-cadherin expression following luteolin treatment in Hs-746T cells, while increased vimentin and pAKT expression. **g** The inhibiting effect of luteolin on cell migration was reversed subsequent to Notch1 overexpressing in Hs-746T cells. **h** Co-IP of β-catenin and NICD in GC cells. The interaction between NICD and β-catenin was abrogated with luteolin treatment in vitro and in vivo. **i** Proposed molecular model for Notch and β-catenin crosstalk. Results are the means of three independent experiments. *P < 0.05, **P < 0.01

To investigate the suppressing effects of luteolin on GC progression whether through regulating Notch1 or not, Notch1 was downregulated or overexpressed in GC cells. Notch1 knockdown in Hs-746T and MKN28 cells decreased the expression of its target genes Hes-1, Hey-1, and cyclin-D1 (Fig. 5a). Moreover, proliferation and migration were inhibited in Notch1-silenced GC cells compared with the control cells (Fig. 5b, c). In addition, Notch1 knockdown promoted cell apoptosis and reversed EMT in GC cells (Fig. 5d, e). However, overexpression of Notch1 recovered EMT in Hs-746T following luteolin treatment as well as elevated AKT phosphorylation (Fig. 5f). The inhibiting effect on cell migration by luteolin treatment was also partially reversed by overexpression of Notch1 (Fig. 5g). These observations confirm that luteolin treatment suppressed GC progression by inhibiting Notch signaling. Furthermore, NICD directly bound with β-catenin to form a complex, while the interaction between NICD and β-catenin was abrogated subsequent to luteolin treatment in vitro and in vivo (Fig. 5h). The interaction between NICD and β-catenin may contribute to promote cell proliferation, cell migration, and inhibit cell apoptosis in GC by regulating downstream target genes (Fig. 5i), which is blocked by luteolin treatment.

### Luteolin suppresses tumor growth in vivo

**Fig. 6:**
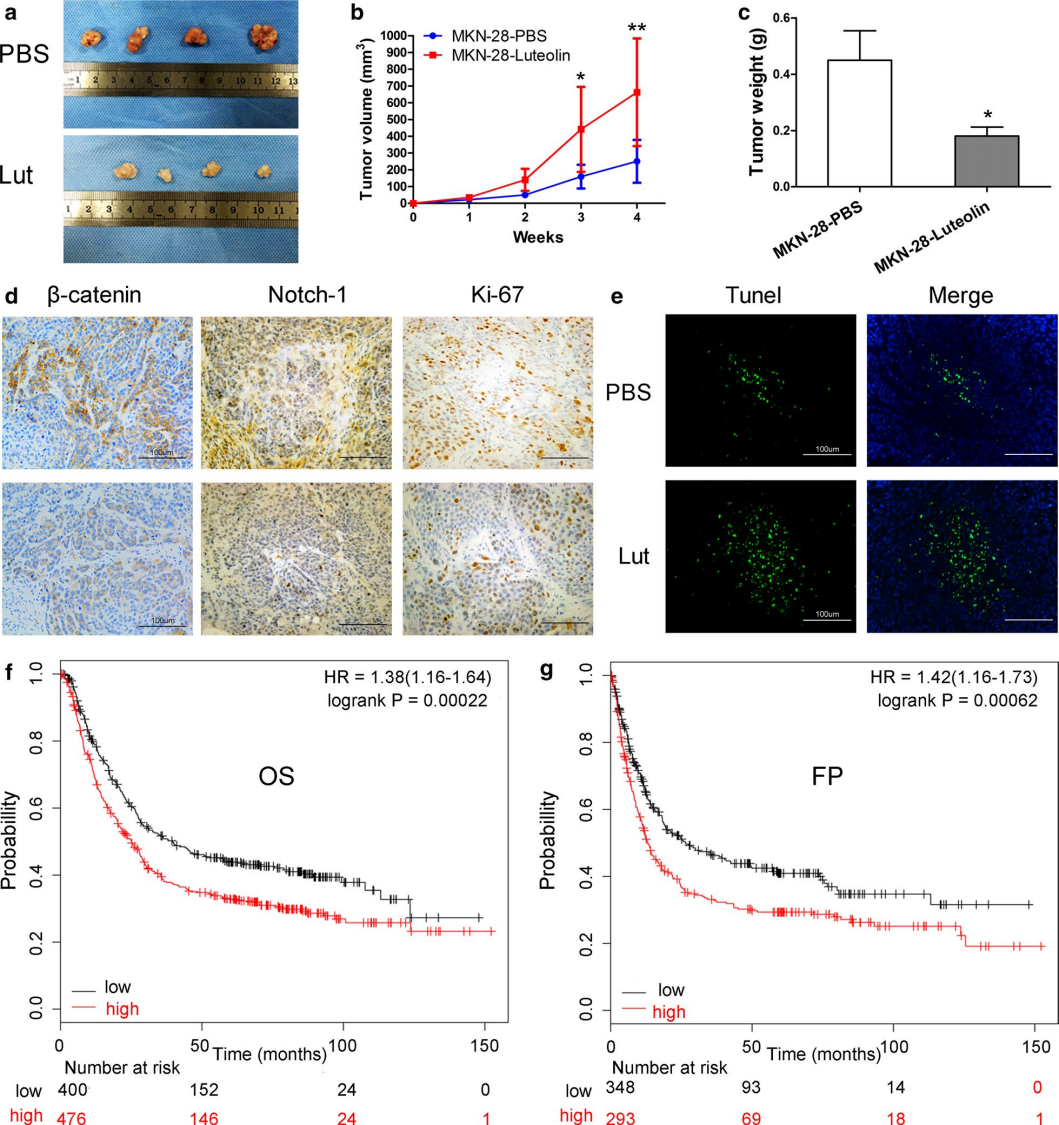
Effect of luteolin on tumor growth in vivo and effect of Notch1 on prognosis. **a** Images of MKN-28 xenograft tumors treated with PBS or luteolin. **b** Tumor volumes were measured every week (*P < 0.05, **P < 0.01). **c** Average weights of xenograft tumors in nude mice (*P < 0.05). **d** Expression of β-catenin, Notch1, and Ki-67 in xenograft tumors by IHC (200×). **e** TUNEL staining of xenograft tumors (200×). **f**, **g** Higher expression of Notch1 was correlated to a poor overall survival (OS) (*P* = 0.00022) and poor time to first progression (FP) (*P* = 0.00062)

To test the effects of luteolin on tumor growth in vivo, MKN28 cells were injected subcutaneously into nude mice. After the tumors were formed, nude mice were injected 6 times intraperitoneally with PBS or luteolin (10 mg/kg). We found that the tumor volume (*P* < 0.01) and tumor weight (*P* < 0.05) in luteolin-treated mice were less than that in the control group (Fig. 6a–c). Furthermore, β-catenin, Notch1 and Ki-67 expression were decreased in tumors from luteolin-treated mice (Fig. 6d); while in contrast, TUNEL staining was elevated in tumors from mice treated with luteolin (Fig. 6e). Analysis of data available online on KMplot indicated that higher expression of Notch1 correlated with a poor overall survival (OS) (*P* = 0.00022, Fig. 6f) and a poor time to first progression (FP) (*P* = 0.00062, Fig. 6g). These results suggest that luteolin can suppress GC progression by inhibiting Notch1 expression.

**Fig. 7:**
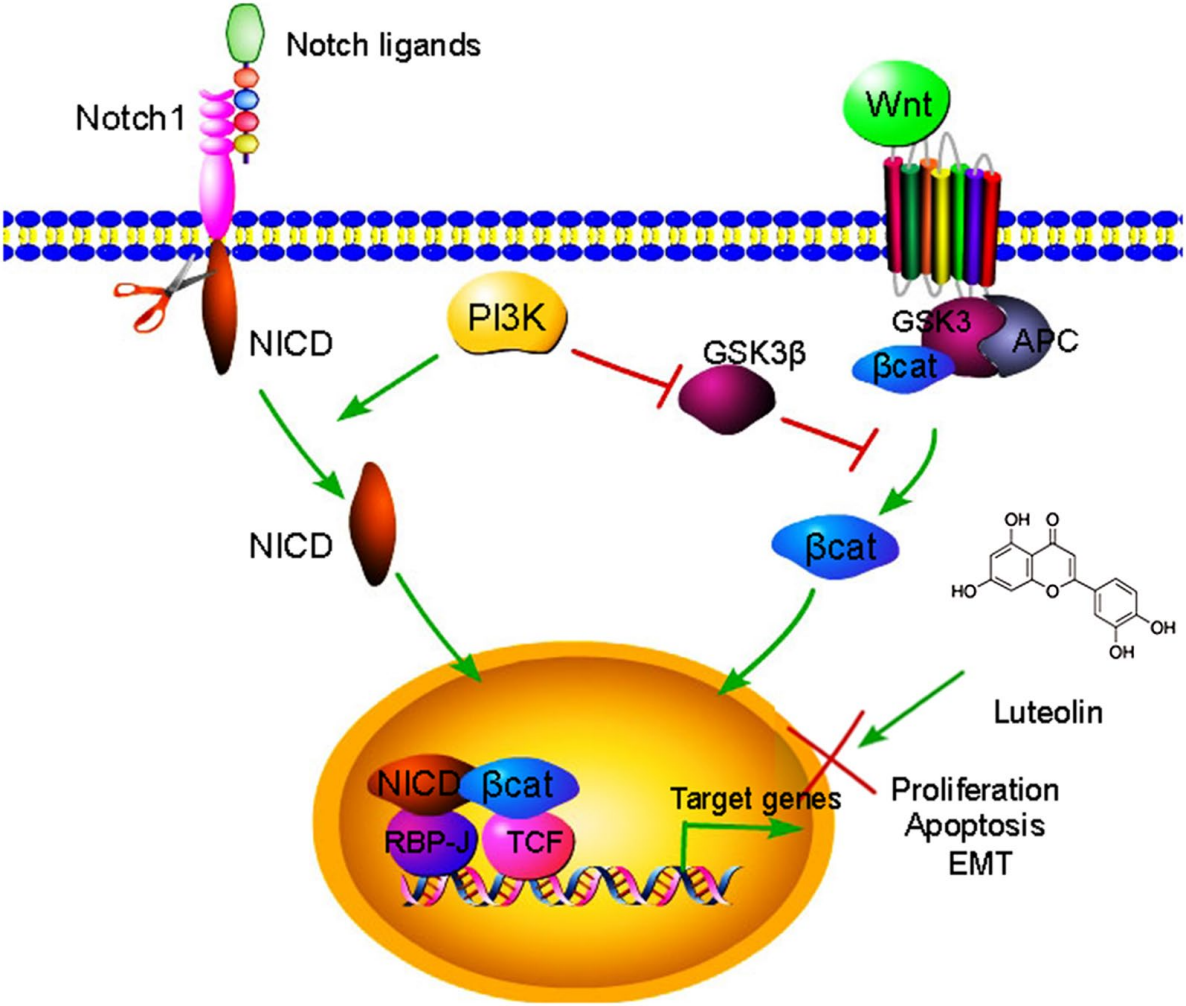
Proposed mechanisms of Notch and Wnt/β-catenin signaling in GC progression. When ligands bind to the Notch receptors, activated NICD translocates into the nucleus and forms a complex with activated β-catenin. The complex formation results in the regulation of target genes to induce cell proliferation and metastasis and inhibits apoptosis. Luteolin blocks the complex formation and inhibits cell proliferation and metastasis, and increases cell apoptosis, suggesting an anti-tumorigenic effect. Luteolin may be a drug for GC treatment

Based on our observations, we propose a model for the roles of luteolin, Notch1, and β-catenin in GC (Fig. 7). Ligand binding activates Notch, causing translocation of NICD to the nucleus, which forms a complex with activated β-catenin. This results in the regulation of target genes and induces cell proliferation and metastasis in GC. The PI3K/Akt signaling enhances the formation of this complex. Luteolin can block Notch signaling, and exerts an anti-tumorigenic effect in GC by inhibiting cell proliferation and migration and increasing cell apoptosis. Thus, luteolin may be an effective drug for the treatment of cancers.

## Discussion

Although tumor resection and chemotherapy contribute significantly to GC treatment, drug resistance and genetic variation reduce their efficacy [32]. A number of compounds obtained from Chinese herbs possess anti-tumor activities, such as those from Taurine [33], Dioscorea bulbifera [34], and Matrine [35, 36]. Although it has been reported that luteolin exerts a marked antitumor effect in cMet-overexpressing patient-derived tumor xenograft models of gastric cancer, luteolin by which way regulating cancer metastasis remains unclear [37]. Luteolin is a flavonoid extracted from Chinese herbs, and in this study, we examined its effects on metastasis in GC and the underlying mechanisms.

Uncontrolled growth of cells is in the hallmark of tumor progression [38]. Hence, we first examined the effects of luteolin on cell proliferation and colony formation in GC, and observed that proliferation of GC cells was significantly inhibited by luteolin in a time- and dose-dependent manner. Next, we found that apoptosis was elevated by luteolin by inhibiting Akt signaling, which itself is implicated in cancer metastasis, proliferation, and apoptosis [39, 40]. We also observed that the cytoskeleton of GC cells shrank upon treatment with luteolin. F-actin staining was used to confirm the changes in the cytoskeleton of GC cells treated with luteolin, and the results suggest that luteolin might inhibit GC motility by reversing EMT in a manner similar to that observed in pancreatic and ovarian cancers [7]. As expected, upon luteolin treatment, the epithelial biomarker E-cadherin was increased. In the contrast, the mesenchymal biomarkers N-cadherin, vimentin, and Snail were reduced in a dose-dependent manner.

Notch signaling was activated during EMT in cardiac development, colorectal cancer, and hepatic carcinoma progression [29, 41]. Notch signaling is predicted to be the potential target of luteolin by our endogenous tumor network model and regulation of Notch signaling plays a key role in tumor progression [42, 43]. Hence, we evaluated the effect of luteolin on Notch signaling, and luteolin treatment indeed inhibited Notch signaling. Silencing of Notch1 inhibited proliferation and migration, induced apoptosis, and reversed EMT of GC cells, which was consistent with the effects observed upon luteolin treatment. Furthermore, overexpression of Notch1 partially recovered EMT and cell migration, illustrating that luteolin suppressed GC progression by inhibiting Notch signaling.

The Notch and Wnt/β-catenin signaling pathways interact in many cell types in synergistic or antagonistic ways, depending on the context [20, 22]; however, it has not been studied in GC. The interaction between β-catenin and NICD was observed in GC cells as well as in leukemia and intestinal stem cells [13, 15], suggesting that they might form a complex to promote GC progression by inducing EMT, elevating proliferation, and inhibiting apoptosis [16, 44]. Luteolin treatment inhibited crosstalk between β-catenin and NICD by decreasing their expression. In an in vivo assay, luteolin suppressed tumor growth by inhibiting proliferation and inducing apoptosis. Luteolin treatment also significantly reduced Notch1 expression. Moreover, higher Notch1 expression was correlated with a poor OS and a poor time to FP [45]. We propose a model for the role of Notch1 and β-catenin in GC progression (Fig. 7). NICD interacts with β-catenin to regulate target genes, causing induction of cell proliferation and metastasis and inhibition of apoptosis. Luteolin inhibits Notch1 and β-catenin expression, thus exhibiting an anti-tumorigenic effect in GC.

## Conclusions

In this study, we found that luteolin significantly suppressed GC progression by inhibiting cell proliferation, migration, and invasion; inducing apoptosis; and reversing the EMT. Meanwhile, the Akt, β-catenin, and Notch signaling pathways were inhibited by luteolin. Furthermore, the effects of downregulation of Notch1 were similar to those observed upon treatment of GC cells with luteolin. In addition, higher Notch1 expression correlated with a poor OS. Luteolin suppressed tumor growth and Notch1 signaling in vivo. Therefore, luteolin may be an effective drug for GC treatment.

## Methods

### Cell culture and materials

The human GC cell lines NCI-N87 and MKN28 were maintained in our lab, and Hs-746T was purchased from American Type Culture Collection. Cells were cultured at 37 °C in 5% CO_2_ and saturation humidity in RPMI-1640 medium with 10% fetal bovine serum containing penicillin and streptomycin. Luteolin was purchased from Aladdin Industrial Corporation (Shanghai). The molecular weight is 286.24 g/mol and the purity is greater than 98% (HPLC).

### Cell proliferation and colony formation assays

Cell proliferation was monitored by Cell Counting Kit-8 (CCK-8). In brief, GC cells were suspended in medium with or without luteolin treatment and then plated in 96-well plate at the concentration 2000 cells/well. Cell proliferation was measured every 24 h for 5 days after adding CCK-8 reagent 2 h at the absorbance 450 nm using Epoch Microplate Spectrophotometer (Bio Tek). For colony formation, Hs-746T and MKN28 cells were plated in 6-well plates at the concentration 1000 cells/well. The experimental groups were treated with luteolin for 24 h groups and then replaced for fresh medium. After 10–14 days, the plates were stained with 1% crystal violet.

### Apoptosis assay

Hs-746T and MKN28 cells were plated in 6-well plates treated with or without luteolin for 24 h. Then cells were collected and examined by apoptosis detection kit (BD Pharmingen). Briefly, 3 μl annexin V-FITC and 5 μl propidium iodide were added into cells successively for 15 min. And then GC cells were monitored by flow cytometry. Right upper quadrant represents percentage of late apoptosis. Right lower quadrant represents percentage of early apoptosis.

### Migration and invasion assays

A number of 1 × 10^5^ GC cells were suspended in serum-free medium with or without Matrigel (BD Bioscience, CA, USA) in upper chambers (Corning Costar, NY, USA) and luteolin or PBS were added into 24-well plates. Next, GC cells were fixed by 10% formalin and stained by 0.5% crystal violet after 24 h. Finally, GC cells that passed through membrane were photographed and counted.

### Immunofluorescent staining

Cells were plated into 8-well glass (Merck Millipore) for overnight and then fixed, permeated and blocked according to protocols. We next stained the cells with β-catenin antibody (1:100; Cell Signaling Technology, CST), followed by incubation with fluorescent secondary antibody for 1 h at room temperature. The nuclei were stained with DAPI. And to visualize the cytoskeleton of GC cells, rhodamine phalloidin (1:20; CST) was used. Slides were analyzed and imaged on a fluorescence microscope.

### Vector construction and transfection

Notch1 shRNA, negative control, pCMV-Notch1 (H3176 pLenti-CMV-MCS-HA-3Flag-P2A-EGFPT2A-Puro), and control vectors were purchased from Oobio Corporation (Shanghai, China) and the vectors carry puromycin-resistance function. siRNA sequences of Notch1 or negative control (NC) were as follows: Notch1, GCAACAGCTCCTTCCACTT; NC, TTCTCCGAACGTGTCACGT. Lip2000 (Invitrogen, Carlsbad, USA) was used to transfect vectors into GC cells and then transfected cells were selected by treatment with puromycin. The effects of shRNA and pCMV-Notch1 were confirmed at protein level using western blot assay.

### Western blot assay

The method was consist with the previous [46]. In brief, proteins of cells were separated by SDS-PAGE and then were transferred into PVDF membranes. Primary antibody (1:1000 dilutions) AKT, p-AKT, NICD, β-catenin, E-cadherin, N-cadherin, Snail and vimentin were purchased from cell signaling technology (CST, USA). Notch-1, Hes-1, Hey-1, Cyclin-D1 (1:1000 dilutions) and GAPDH (1:10000 dilutions) were purchased from Proteintech. After incubation with primary antibody, secondary antibody followed. Finally, the results were visualized by Tanon system.

### Immunoprecipitation experiment

Cells were lysed at 4 °C using RIPA and the followed procures were based on Co-IP kit (Pierce, Rockford, USA) according to manufacturer’s instructions. In brief, a total of 300 μg proteins were incubated overnight with specific primary antibodies β-catenin and NICD at 4 °C. Immune complexes were precipitated with protein A/G Sepharose beads and next were examined by western blot.

### RT-PCR assay

RNA was extracted from GC cells treated with luteolin or without, and then RNA was reversed to cDNA. Primers for Notch-1, forward GCTTGTGGTAGCAAGGAAGC (20b), reverse CCACATTCAAGTGGCTGATG (20b); Hes-1, forward ACACGACACCGGATAAACCAA (21b), reverse CGAGTGCGCACCTCGGTA (18b); Cyclin-D1, forward GGGTGGGTTGGAAATGAACT (20b), reverse CTTCCTCTCCAAAATGCCAG (20b). RT-PCR was performed using SYBR-green according to manufacturer’s instructions.

### In vivo experiment and immunohistochemistry

BALB/c male nude mice (Institute of Zoology, China Academy of Sciences) were used to evaluate the role of luteolin in tumor growth in vivo. Nude mice received humane care and the study protocols were carried out according to a protocol approved by the Institutional Animal Care and Use Committee (IACUC) at Shanghai Jiao Tong University, Shanghai, China. Tumor nodules were measured every week, and was calculated using formula: tumorous volume = (width^2^ × length)/2. Mice were killed at 4 weeks after injection and then tumors were weighed and fixed for immunohistochemistry staining (IHC).

For IHC, sections staining was performed according to the DAKO protocol, using primary antibody (1:200 dilutions) Notch1, β-catenin and Ki-67. The tunel assay was performed using In situ cell death detection kit (Roche). Blue represents nucleus, green represents death cells.

### Statistics

Differences between experimental groups were assessed by the Student’s t test or one-way ANOVA. Student’s *t* test was used to examine the statistical differences between the two groups. The significant inhibited effect on cell growth by luteolin was observed at 4th and 5th day after luteolin treatment. The results of 4th and 5th day were compared to that in their control groups using the Student’s t test in Fig. 1a, as well as in Fig. 1b. Survival was analyzed with the Kaplan–Meyer method comparing survival curves by log-rank test. Data are shown as mean ± SD. A two-tailed value of *P* < 0.05 was considered statistically significant. Statistical analyses were performed using IBM SPSS 19.0 software (SPSS Inc).

## Abbreviations

GC: gastric cancer
NICD: Notch intracellular domain
CCK-8: cell counting kit 8
RT-PCR: real-time reverse transcription polymerase chain reaction
EMT: epithelial-mesenchymal transition
Co-IP: co-immunoprecipitation
OS: overall survival
FP: first progression
GAPDH: glyceraldehyde-3-phosphate dehydrogenase
